# The genome of the reef-building glass sponge *Aphrocallistes vastus* provides insights into silica biomineralization

**DOI:** 10.1098/rsos.230423

**Published:** 2023-06-21

**Authors:** Warren R. Francis, Michael Eitel, Sergio Vargas, Catalina A. Garcia-Escudero, Nicola Conci, Fabian Deister, Jasmine L. Mah, Nadège Guiglielmoni, Stefan Krebs, Helmut Blum, Sally P. Leys, Gert Wörheide

**Affiliations:** ^1^ Department of Earth and Environmental Sciences, Paleontology and Geobiology, Ludwig-Maximilians-Universität München, Munich, Germany; ^2^ Laboratory for Functional Genome Analysis (LAFUGA), Gene Center, Ludwig-Maximilians-Universität München, Munich, Germany; ^3^ GeoBio-Center, Ludwig-Maximilians-Universität München, Munich, Germany; ^4^ Department of Biological Sciences, University of Alberta, Edmonton, Canada T6G 2E9; ^5^ Service Evolution Biologique et Ecologie, Université libre de Bruxelles (ULB), 1050 Brussels, Belgium; ^6^ Staatliche Naturwissenschaftliche Sammlungen Bayerns (SNSB)–Bayerische Staatssammlung für Paläontologie und Geologie, Munich, Germany

**Keywords:** sponge, Porifera, Hexactinellida, genome, silica, differentially expressed genes

## Abstract

Well-annotated and contiguous genomes are an indispensable resource for understanding the evolution, development, and metabolic capacities of organisms. Sponges, an ecologically important non-bilaterian group of primarily filter-feeding sessile aquatic organisms, are underrepresented with respect to available genomic resources. Here we provide a high-quality and well-annotated genome of *Aphrocallistes vastus*, a glass sponge (Porifera: Hexactinellida) that forms large reef structures off the coast of British Columbia (Canada). We show that its genome is approximately 80 Mb, small compared to most other metazoans, and contains nearly 2500 nested genes, more than other genomes. Hexactinellida is characterized by a unique skeletal architecture made of amorphous silicon dioxide (SiO_2_), and we identified 419 differentially expressed genes between the osculum, i.e. the vertical growth zone of the sponge, and the main body. Among the upregulated ones, mineralization-related genes such as glassin, as well as collagens and actins, dominate the expression profile during growth. Silicateins, suggested being involved in silica mineralization, especially in demosponges, were not found at all in the *A. vastus* genome and suggests that the underlying mechanisms of SiO_2_ deposition in the Silicea *sensu stricto* (Hexactinellida + Demospongiae) may not be homologous.

## 1. Introduction

Reefs are geobiological structures formed by organisms that elevate themselves above the seafloor by forming a skeleton in a process called biomineralization, i.e. the biologically controlled formation of composite materials [1]. While today extensive reefs are mainly built by scleractinian corals using calcium carbonate in shallow tropical seas, during the Late Jurassic siliceous hexactinellid (glass) sponges built substantial bioherms forming significant reef communities in moderate (mesophotic) water depths on the northern Tethys shelf in Europe [2]. In fact, during that time, siliceous sponges formed the largest reef belt ever built in Earth's history, scattered over more than 7000 km and extending into the North Atlantic Basins [3].

Hexactinellida, or glass sponges, are one of the four classes of Porifera (sponges) [4]. They have a number of unusual characteristics compared to other sponges, foremost among which are the syncytial nature of most of their cells, the ability to propagate electrical signals to arrest their feeding current, and their most well-known feature, a unique and often massive, skeletal architecture made of amorphous silicon dioxide (SiO_2_) [5]. How silica is deposited in cold water and in such quantities is still poorly understood [6,7]. Hexactinellida is the sister group to the Demospongiae that also, with some exceptions, secrete SiO_2_ skeletal elements. The main model of Demospongiae biosilification involves silicateins and cathepsins [8], but silicateins are thought to be absent from glass sponges, where other proteins are used instead [7,9]. Demosponges can be collected in shallow waters, and some can be kept in the laboratory, which has significantly advanced the study of that group. By contrast, hexactinellids are mainly found in the deep sea with considerable undocumented species diversity [10]. A few species, however, occur in shallower water, and of these three species form globally unique reef structures on the coast of British Columbia (BC, Canada) [11,12]. These reefs are vast, covering more than 400 km^2^ in 240–100 m water depth; they rise up to 20 m above the seafloor and are often many kilometres wide [3]. Locally, they are of great ecological and biogeochemical importance [13,14], but are threatened by anthropogenic activities, and several are the focus of newly formed Marine Protected Areas [15,16]. A key attribute for building a reef is fast growth upward to keep above the sedimentation that is naturally baffled by the reef structure and which is necessary for cementing the reef framework at its base. Reef forming sponges also use secondary silicification to fuse the framework into a three-dimensional scaffold. As a result, reef forming sponges are excellent models for understanding new skeleton formation in hexactinellid sponges.

**Figure 1.**
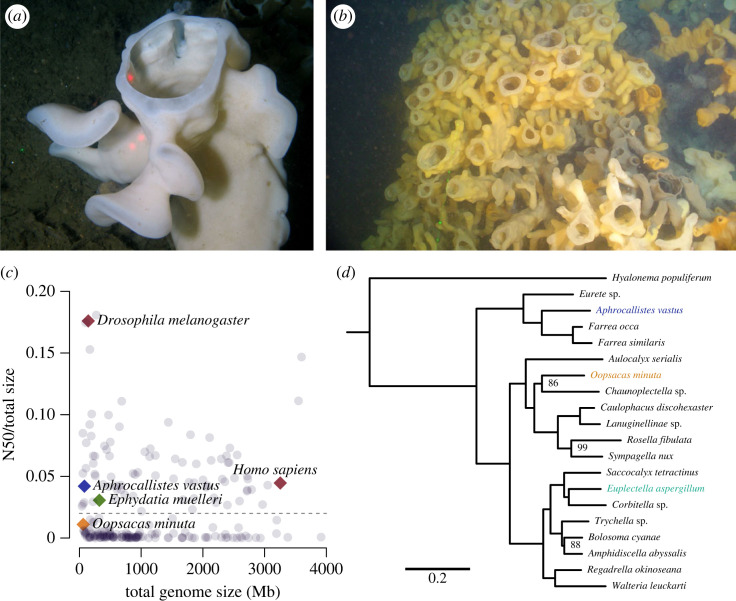
*Aphrocallistes vastus*: habitus, genomic overview and phylogenetic grouping in the Hexactinellida. (*a*) Photograph of *Aphrocallistes vastus* at 170 m depth on the Hecate Strait and Queen Charlotte Sound glass sponge reefs. Lasers top to bottom right are 10 cm apart (photo by James Pegg, the ROV pilot). The single red laser dot marks the oscular region (‘tip’) and the two laser dots the main ‘body’, the two regions from which differentially expressed genes were assessed. (*b*) Photograph taken by ROV of the sponge reefs at Fraser Ridge in the Salish Sea, BC, Canada. Oscula (round openings) are about 5 cm in diameter. (*c*) Assembly N50 over total size. Chromosome-level assemblies should count around 0.02 or higher (dashed line), as most of the N50 would be represented by chromosome-sized pieces for typically 10–30 chromosomes (electronic supplementary material, table S7). (*d*) Multigene phylogeny of glass sponges, tree rooted with the demosponges *Amphimedon queenslandica* and *Ephydatia muelleri* (not shown). All nodes have 100/100 bootstrap support unless otherwise noted. This phylogenetic inference of BUSCO orthogroups is consistent with the current hypothesis of hexactinellid relationships (e.g. [19]).

One of the key species in the BC sponge reefs is the hexactinosan hexactinellid *Aphrocallistes vastus* Schulze 1886, the ‘cloud sponge’, a species that also occurs in shallower waters in fjords in BC (as shallow as 15 m) [17]. This species can grow up to two metres in size, but its body wall is fragile with ‘a texture and friability of a thin slice of toast’ [18]. *A. vastus* has a distinctive morphology with palm-shaped folds arising from the side of the body, and upward growth taking place at the osculum where soft tissues first arise, and new feeding chambers and spicules are formed (figure 1*a*,*b*) [20]. *A. vastus* is an emerging glass sponge model species because many aspects of its ecology, morphology, and ultrastructure have been studied previously [20] (e.g. [21,22]). To fully take advantage of this model for understanding the mechanisms underlying silica biomineralization, information from the nuclear genome is needed.

Here, we present the draft genome of the reef-building glass sponge *Aphrocallistes vastus,* to address questions of particular relevance for the Hexactinellida, such as the formation of their glass spicules. We take advantage of the recent publication of genome data of another glass sponge, *Oopsacas minuta* Topsent 1927, a small, non-reef-forming hexactinellid found in Mediterranean caves [23]. The genome of *O. minuta* is small (61 Mb), highly compact, and is assumed to be secondarily lacking key genes involved in morphogenetic processes (such as *wnt*, *wntless*, or *dishevelled*) that are considered to be ancestral to all metazoans. These findings raise important questions about the gene content and genomic architecture of glass sponges, in particular genome rearrangements, chromosome loss, and the tempo and mode of genome evolution in this group. Our analysis of the draft genome of *A. vastus* focuses on genome gene content, architecture and synteny to provide first insights into the general properties and architecture of the genome of glass sponges. To address the genomic mechanisms responsible for the glass spicule formation that enables this species to grow tall and form reefs, we analysed differentially expressed genes between two different regions of the sponge: the oscular region, where new tissue and spicules necessary for vertical growth are formed, and the older body parts where this process does not take place. Our results confirm that glass sponge genomes are small, compact, with a large number of nested genes, and a high degree of microsynteny. We show that growth zones of this reef-forming species are characterized by high levels of expression of structural genes. Overall our results provide deeper insights into the genome architecture of hexactinellids and their potential for adaptation to environmental change that has driven their unusual cellular, physiological and skeletal characteristics.

## 2. Results and discussion

### 2.1. The *A. vastus* genome is small compared to most metazoans

With a hybrid sequencing strategy, we sequenced and assembled the genome of *Aphrocallistes vastus* to 112x coverage (electronic supplementary material, figure S1). The final 80 megabase genome assembly contained 186 scaffolds with an N50 of 3.3 Mb (figure 1*c*). A total of 27 scaffolds larger than 500 kb comprised 96% of the genome, indicating that many of these scaffolds are likely to be large pieces of chromosomes, making the chromosome number likely to be 10–12n (electronic supplementary material, figure S2). The small *A. vastus* genome has many features of other small genomes: dense gene arrangement (electronic supplementary material, figure S3), short introns, and a large number of single-exon genes (table 1). In fact, the largest protein in the genome, Avas.s016.g412.i1, is a 49 014 amino acid protein of a predicted mass of 5.34 MDa, encoded by a single 147 kbp exon. The average *A. vastus* gene length (approx. 2.5 kb) is small for an animal, but still over double that of a typical bacterial gene.

**Table 1.** Comparison of broad genome features among representative sponge genomes.

species	genome size (Mb)	no. scaffolds	genes	no. single exon transcripts	no. nested genes identified	version
*Aphrocallistes vastus*	80.32	186	19 578	12 409	2432	v1.29
*Oopsacas minuta*	61.46	365	16 340	9872	83	v1
*Ephydatia muelleri*	322.62	1444	39 245	7075 (75)^a^	0	v1
*Amphimedon queenslandica*	166.67	13 397	43 615	13 201	3358	v2.1
*Sycon ciliatum*	357.50	7780	32 309	17 172	5032	v1

^a^Refers to different versions of the annotation, as one version required a splice site and excluded most single exon genes.

During our initial assembly, 22 bacterial scaffolds were identified. These scaffolds were collected into two bins, with one of the bins containing a single 1.5 Mb scaffold. The two bacteria were identified as belonging to alpha- and beta-proteobacteria but were not analysed further. As *A. vastus* was not thought to harbour a high diversity of bacterial symbionts (with only one bacterium reported by [5]), the nature of the association with the microbes is uncertain.

We manually annotated the genome based on the gene models from BRAKER2 [24,25], which were then substituted by other transcripts or gene models based on the appraisal of the annotator. This yielded 19 578 genes, of which 16 416 transcripts were kept unchanged from the BRAKER2 models (see electronic supplementary material, tables S3 and S4). Thus, about one in five genes required some manual correction. The final gene count is substantially fewer than that of other sponges (table 1), but slightly more than the genome of the recently released glass sponge *O. minuta* [23]. As with other non-model genomes, many genes are difficult to annotate due to low coverage or apparent lack of homologues.

From the sequencing of long cDNA reads, we were able to identify an approximately 37 bp trans-spliced leader sequence motif (electronic supplementary material, figure S4). The sequence of the trans-spliced leader itself differed from those found in other animal phyla [26–30], though suggests that mRNA trans-splicing is ancestral, and possibly necessary, among animals. In the ctenophore *Hormiphora californensis*, half of the reads had a skipped portion at the 5′ ends from mapping, of which the most common sequence accounted for 55% of the detected leader sequences. However, in *A. vastus*, only 4% of the sequenced long reads had predicted leader sequences, and many more different leader sequences were identified among those reads. That is, the most common leader sequence only accounted for around 2% of all the detected leaders, likely as a result of the comparatively high error rate of NanoPore sequencing compared to PacBio (used for *H. californensis*). Our annotation strategy also identified 2432 nested genes, i.e. those contained in an intron of another gene (table 1). The fraction of nested genes in *A. vastus* (12.4%) is larger than that reported in other genomes, which typically ranges between 5 and 10% of all genes, but is almost zero in several genomes [27]. The low number of nested genes detected in some sequenced genomes is probably caused by a disabled option in some gene modelling programs and highlights the substantial effect of the annotation method on the quality of the final gene set.

### 2.2. Extensive microsynteny is found among glass sponges

**Figure 2.**
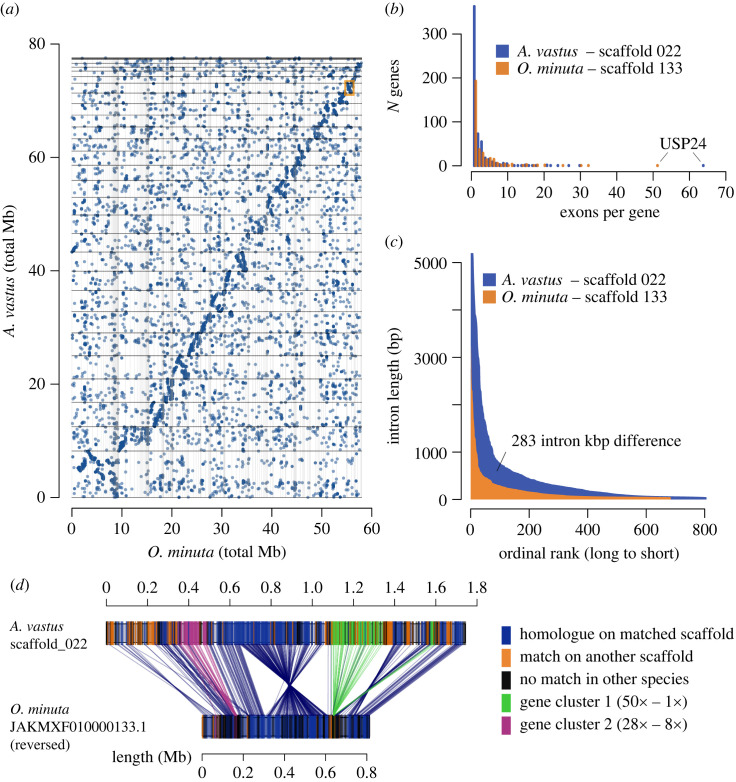
Synteny between long scaffolds. (*a*) Dot plot between the two glass sponges *A. vastus* and *O. minuta*, where each point represents a gene match between the two species. There is a general trend of synteny between the two, visible as diagonal lines within each pair of scaffolds (see electronic supplementary material, figure S5, for higher resolution). (*b*) Histogram of exons per gene, for both species. The largest two non-syntenic gene clusters are highlighted in green and purple, respectively. Gene USP24 varies in exon number due to misannotation in *O. minuta* (see electronic supplementary material, Alignment 1 in the project's GitHub repository at https://github.com/PalMuc/Aphrocallistes_vastus_genome). (*c*) Lengths of all introns on *A. vastus* scaffold 022 and *O. minuta* scaffold 133, ordered from longest to shortest, showing the predominance of longer introns in *A. vastus*. (*d*) Synteny diagram between two scaffolds of *A. vastus* (022, top) and *O. minuta* (133, bottom).

We then compared the genome structures of *A. vastus* and *O. minuta* (for a phylogenetic relationship of *A. vastus, O. minuta* and the additional hexactinellids included in this study, see figure 1*d*). Although neither assembly was to the level of chromosomes, we nonetheless found 848 total putative synteny blocks of three genes or more, accounting for 8106 genes. Thus, nearly half the total genes of each species are syntenic. Many of these blocks appeared to span entire scaffolds of *O. minuta* (figure 2*a*), showing that chromosomal inversions were relatively uncommon in these two lineages. Compared to ctenophores [27], which show rapid rearrangement of the genome between species, it appears that the subclass Hexasterophora, to which both *Aphrocallistes* and *Oopsacas* belong, have had very few genomic rearrangements. Either the radiation of hexasterophoran hexactinellids is much more recent, or other biological factors control the frequency of inversions or translocations in this group.

The relatively long scaffolds of *A. vastus* and the manual annotation of its genes allowed us to examine two key unresolved issues—what directs inversion breakpoints, and where do novel genes that are not syntenic come from? One example of homologous scaffolds is *A. vastus* scaffold 022 with 544 genes (568 transcripts) matching *O. minuta* scaffold 133 with 340 genes (figure 2*d*). The *A. vastus* scaffold is almost double the length of the *O. minuta* scaffold but still incorporates 248 homologous genes in synteny, except for a few large inversions. The size difference between these scaffolds can be accounted for by the addition of more single exon genes (figure 2*b*) and larger introns in *A. vastus* (figure 2*c*). In this regard, the scaffold of *O. minuta* has 684 unique introns with an average size of 194 bp and a total (sum) length of 133 kb, while the scaffold in *A. vastus* has 806 introns of 516 bp on average and a total (sum) length of 416 kb, almost triple that of *O. minuta*. Unsurprisingly, the average transcript length is almost the same (1429 bp and 1426 bp, for *O. minuta* and *A. vastus*, respectively) suggesting that most genes in the syntenic block are orthologues and have not changed appreciably in size in both lineages.

We then examined the 204 putative non-syntenic genes on the *A. vastus* scaffold. These genes tended to occur in clusters of tandem duplications, likely representing gene family expansions specific to *A. vastus*. Three larger clusters accounted for 92 total genes on scaffold 022, though meaningful functional predictions could only be made for one of them. The largest cluster of the three contains 75 protein-coding genes from all *A. vastus* scaffolds, where 50 of the protein-coding genes come from a tandem block on scaffold 022 (figure 2*d*, gene cluster 1 in green). Of these 50 genes, 38 are single exon genes, although this may be an underestimate due to difficulties in mapping RNA to tandem duplicates. Based on the observed synteny, *O. minuta* gene LOD99_16049 is the only gene in the syntenic position on the corresponding scaffold, indicating that the *A. vastus* cluster on scaffold 022 is an expansion unique to *A. vastus*, potentially starting from duplications of gene Avas.s022.g348.i1. The mechanism leading to the expansion is uncertain, as the genes are not in strict synteny (they are interleaved with genes that have homologues on other scaffolds, and they are all single exon in both species, making both replication slippage and retrotransposition possible. Functionally, these proteins contain AIG1 domains, a domain found across eukaryotes and thought to be involved in pathogen recognition in plants [31]. Expansions of genes involved in the immune system have been identified in the non-syntenic regions between haplotypes in the pearl oyster [32], and could be common among invertebrate genomes. These gene clusters are also roughly at the breakpoints for the chromosomal inversions observed between this scaffold and *O. minuta* scaffold 133. However, none of the breakpoints appears to be directly flanked by the gene clusters, suggesting that the expansion at these loci may be a consequence of other yet-to-be-identified genomic changes.

Of the remaining 110 genes without syntenic orthologues, three were excluded, leaving 107 genes. Of those, 42 were not included in orthologue groups by our clustering, while 52 were in paralogous protein clusters, and 13 were in hexactinellid-specific orthologue clusters lacking functional predictions. Only four of the 52 genes matched retrotransposons or reverse transcriptase/integrase genes. As 83 of the 110 genes were single exons, the insertion of novel genes by retrotransposition may be a primary mechanism of breaking synteny.

### 2.3. Genes involved in the mineralization of silica

Biomineralization systems appear to have evolved largely convergently in the different clades of organisms. Still, among all animals silica is only produced in considerable amounts by siliceous sponges [33], and among those, most prominently in the class Hexactinellida (glass sponges). It is this capacity that enables Hexactinellida to build massive reefs today in British Columbia. The Hexactinellida is named after the presence of 6-rayed silica spicules in an octahedral/triaxonic shape [4,34] in their skeletons, and while the highly hierarchical architecture of the glass sponge skeleton and its use for biomimetic material production are well-acknowledged [35], the molecular mechanism leading to silica mineralization in this group is incompletely understood, in that necessary and sufficient gene sets have not been identified. In class Demospongiae, silicatein is one of the main enzymes apparently involved in biosilica deposition, and although earlier studies reported this enzyme to also be present in Hexactinellida (*Crateromorpha meyeri*) [36], no silicateins were subsequently found in glass sponge transcriptomes [37] nor the *O. minuta* genome [23]. We also did not identify any silicateins in *A.vastus*, hence earlier findings likely represent contaminations.

While different clades of organisms might not share key genes for biomineralization, there are many functional similarities between different mineralization systems: an organic scaffold, typically with alternating polar or charged residues (such as the amino acids DS, in the pearl oyster [38]), and accessory enzymes to process the inorganic ions (such as removal of H_2_O by carbonic anhydrase). In fact, some key genes, such as carbonic anhydrases, appear to be homologous and broadly used for biomineralization [39,40]. Proteins functioning as scaffolds for biomineralization are united by having an amino acid compositional bias and many regions of intrinsic disorder, and often will not align with each other, nor will they be found by the BLAST algorithm due to their low complexity [41]. Highly repetitive proteins present significant challenges for both X-ray crystallography (for instance, difficulties in crystallizing gluten proteins [42]) and *in silico* structure prediction due to intrinsically disordered regions (for instance, see the Alphafold prediction of human dentin: https://alphafold.ebi.ac.uk/entry/Q9NZW4). Thus, alternative strategies may be required to identify novel biomineralization proteins in new taxa.

**Figure 3.**
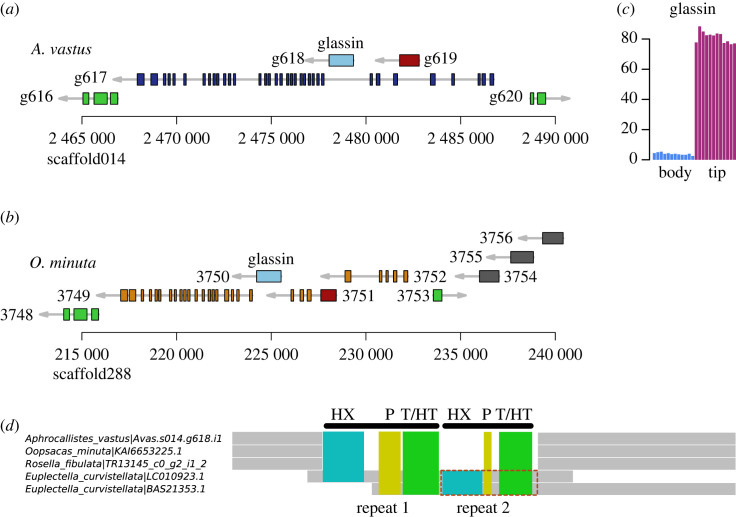
Glassin locus and expression. (*a*) 25 kbp span of the glassin locus in both species. The red gene Avas.s014.g619 is nested in *A. vastus* but the orthologue is annotated as a starting exon in *O. minuta* for another gene. (*b*) The homologous glassin locus in *O. minuta*. (*c*) RNAseq expression counts of the glassin gene in the developing osculum (‘tip’) compared to the main ‘body’. (*d*) Schematic alignment of glassin proteins (see electronic supplementary material, figure S6, or electronic supplementary material, Alignments 2 and 3 in the GitHub repository at https://github.com/PalMuc/Aphrocallistes_vastus_genome for additional hexactinellid glassins identified in the newly generated transcriptomes). The grey regions are well-aligned between the sequences. The 6 repetitive regions are coloured, but align poorly between the species as the repeats are different, and likely rapidly mutating. The red box indicates an overlapping region between the two *E. curvistellata* transcripts that differ at the sequence level, suggesting the presence of at least two genomic loci in that species.

One gene thought to be involved in silica biomineralization in hexactinellids is glassin, a novel histidine-rich protein containing HX or HHX repeats [9,43]. Considering this, we first searched for homologues of *Euplectella* glassin in the *A. vastus* genome and were able to identify a complete orthologue of this gene both in this (Avas.s014.g618) and in the *O. minuta* genome (LOD99_3750). In both genomes, glassin is encoded in a single exon and is nested inside another gene (figure 3*a*,*b*). The predicted proteins are estimated to be around 48 kDa for both species, double the size of the 23 kDa glassin protein identified by SDS-PAGE from *E. curvistellata* [9]. This suggests that the pro-glassin protein is cleaved into the final form by an unknown protease. Several similar EST sequences of glassin were originally identified, though only one of the sequences is clearly an orthologue of the single full-length glassin locus in both *A. vastus* and *O. minuta*. As this gene is a single exon, variant proteins would not be produced by alternative splicing. We were unable to find any paralogue of this gene in the *A. vastus* genome (or alternative annotations), suggesting that *E. curvistellata* and other glass sponges may have additional copies in those lineages. The partial *E. curvistellata* glassin proteins are highly repetitive with HD and TH repeats; however, outside of the conserved C-terminal sequence, these regions poorly align with the repeats from other species (figure 3*d*), suggesting rapid accumulation of substitutions in this gene. Additionally, we could find homologues of glassin in most of the transcriptomes of other glass sponges. These proteins shared the same features: a highly conserved C-terminal domain, and a series of histidine, proline and serine/threonine repeats that are mostly species-specific.

**Figure 4.**
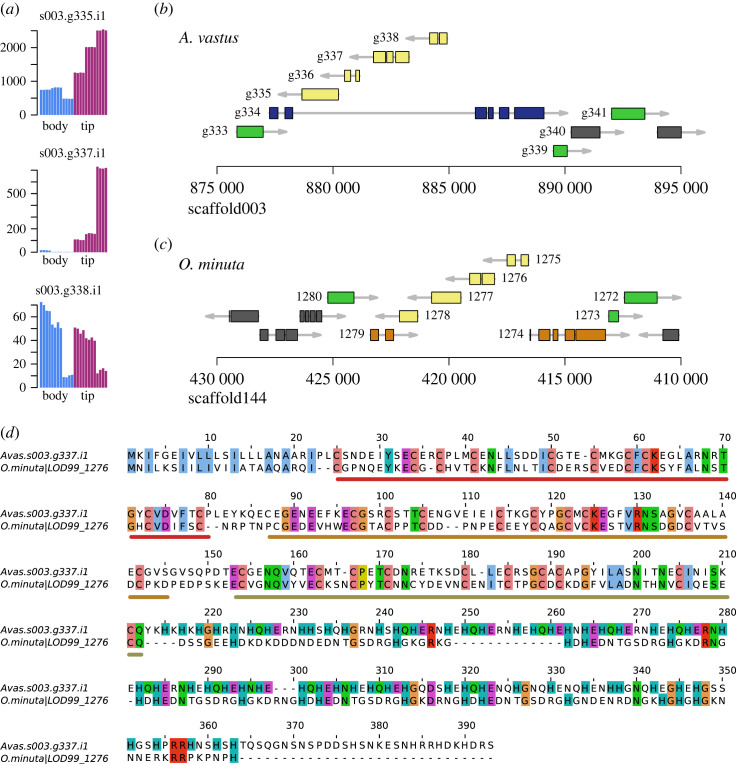
TIL-domain proteins—candidate matrix scaffolding proteins used in silica mineralization. (*a*) Expression profiles of the 3 TIL-domain proteins with s003.g337.t1 being the best scaffolding protein candidate since it is significantly upregulated in the developing osculum (tip). (*b*) 20 kb span of the loci of the TIL-domain proteins, shown in yellow. Green genes have syntenic orthologues in *O. minuta*. (*c*) 20 kb span of the corresponding locus for *O. minuta*. Note that the scaffold is reversed to show synteny. (*d*) Alignment of s003.g337 with the *O. minuta* orthologue LOD99_1276. The three TIL domains characterized by 10 cysteines are shown as red, orange and brown lines under the alignment. After this, the alignment is poor and consists mostly of histidine-rich repeats.

As glassin may be one of the possibly many scaffolding proteins used by hexactinellids for silica mineralization, we searched for proteins with compositional bias, that is, when the frequency of any amino acid was in the top 99th percentile relative to all of the proteins in SwissProt. This identified 330 proteins. As the amino acids DEHPST are frequently used in proteins involved in biomineralization in other animal phyla and in diatoms [38,44,45] (e.g. [46]), we narrowed our search to proteins enriched in those amino acids, without a known function (e.g. excluding collagen), and significant expression in the developing sponge osculum as biomineralization is active in this part of the sponge body (electronic supplementary material, tables S8). We identified a candidate histidine-rich protein that was upregulated approximately 50-fold in the developing osculum (tip) when compared to the main body (body) (figure 4*a*). The protein, Avas.s003.g337.i1, contains three trypsin-inhibitor-like (TIL) domains as well as an approximately 120AA stretch of alternating charged residues, approximated as HEH(N/Q) (figure 4*d*). The biological function of this protein is uncertain, and although it has some characteristic features of mineralization proteins, we could not investigate this biochemically. Based on the synteny (figure 4*b*,*c*), the orthologue of this gene in *O. minuta* is LOD99_1276. In both species, these genes are found in a tandem group of four related genes, with related genes found in the transcriptomes of other glass sponges (shown in electronic supplementary material, Alignment 3). One of them, Avas.s003.g336.i1, does not appear to make a complete protein. Of the four genes, only Avas.s003.g337.i1 is significantly upregulated in the developing tip (*p*-adj: 1.8 × 10^−6^) suggesting that the transcription of these four genes is not coregulated.

**Figure 5.**
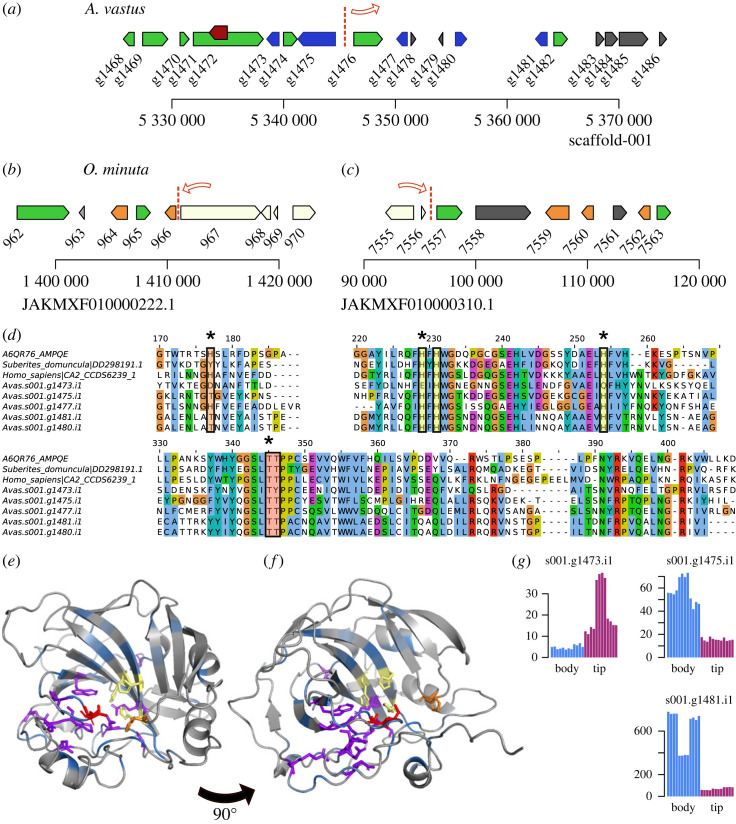
Carbonic anhydrase locus. (*a*) 50 kb span on *A. vastus* scaffold 001 showing the 5 copies of carbonic anhydrases in blue. Other syntenic genes are shown in green, while non-syntenic genes are shown in dark grey. The red line indicates where the synteny block breaks between *O. minuta* scaffolds 222 and 310. (*b*) The corresponding locus in *O. minuta* for two of the five carbonic anhydrases is shown in orange. Genes shown in white are located on a different part of *A.vastus* scaffold 001 corresponding to genes s001.g1342 through s001.g1378, indicating one or more inversions have taken place. (*c*) The corresponding locus for the other 3 carbonic anhydrases in *O. minuta*. (*d*) Multiple sequence alignment of carbonic anhydrases, only showing blocks of the binding pocket residues. (*e*) Modelled structure of a putative carbonic anhydrase from *A. queenslandica* from (*d*), looking into the binding pocket, and (*f*) the same structure rotated 90 degrees. Yellow residues show the triad of histidines to coordinate zinc; red residues show the threonine pair to coordinate the CO_2_; and the orange histidine is a proton acceptor from water. Other residues are coloured based on percent identity. Purple residues indicate 100% identity, mostly beneath the binding pocket. None of the 6 active site residues are 100% conserved. (*g*) Normalized RNAseq expression counts. Adjusted *p*-values are 3.8 × 10^−4^, 6.7 × 10^−7^ and 1.0 × 10^−12^ for g1473, g1475 and g1481, respectively. The expression of the other two paralogues is not significantly different between the body and tip.

Silicase is a hydrolase related to α-carbonic anhydrases [47] that was described as being bifunctional for carbonic anhydrase and silica hydrolase activity. Four paralogues were found in *O. minuta* [23]. We had identified five in *A. vastus* forming a syntenic block with those in *O. minuta* (figure 5*a–c*), also revealing an additional copy in *O. minuta*. These sequences formed a glass-sponge specific clade of four orthologues, with one clade having independent duplications in both *A. vastus* and *O. minuta* (see electronic supplementary material, tree 2). The α-carbonic anhydrase active site consists of a triad of histidines that bind a zinc ion (H94, H96, and H119 in human CAII), two threonines for coordinating carbon dioxide (T198 and T199 in human CAII), and a histidine (H64 in human CAII) needed for a proton transfer from water [48]. While this position is tyrosine in the *S. domuncula* silicase, other animal homologues have a variety of amino acids at this position, including Y in mammal CA5a/b, R or K in several mammal CAs, and S, N or Q in some other species. The many possible H64 replacements do not clearly correspond with changes in the activity in different clades. In the glass sponges (electronic supplementary material, figures S5*d–f* and S7), only one of the four clades has a histidine at this position, and most sequences instead have threonine. One paralogue in particular, Avas.s001.g1473.i1, has substitutions in 4/6 of the active site residues. This paralogue was upregulated in the tip of one of the biological replicates (figure 5*g*). While the substitutions could change the function, it was demonstrated that the carbonic anhydrase/silicase from *S. domuncula* also possessed carbonic anhydrase activity [47], and was suggested to affect silicification by also changing the pH.

### 2.4. Structural genes are upregulated in the growing osculum

We then broadly examined genes that are differentially expressed between the developing osculum, i.e. the vertical growth zone of *A. vastus*, and the older ‘body’ (‘tip’ versus ‘body’ in figure 1*a*). It has been shown by EdU labelling that new cells are only added in this growing ‘tip’ [20] and we specifically sampled this region and compared it to the control ‘body’ region. A total of 419 genes were significantly up- or downregulated, with 342 upregulated and 77 downregulated (counted using an adjusted *p*-value threshold of 10^−4^; see electronic supplementary material, figures S8 and S9, or electronic supplementary material, tables S8 and S9). Scaffold 012 did not appear to have any differentially expressed genes, although it is not clear why.

**Figure 6.**
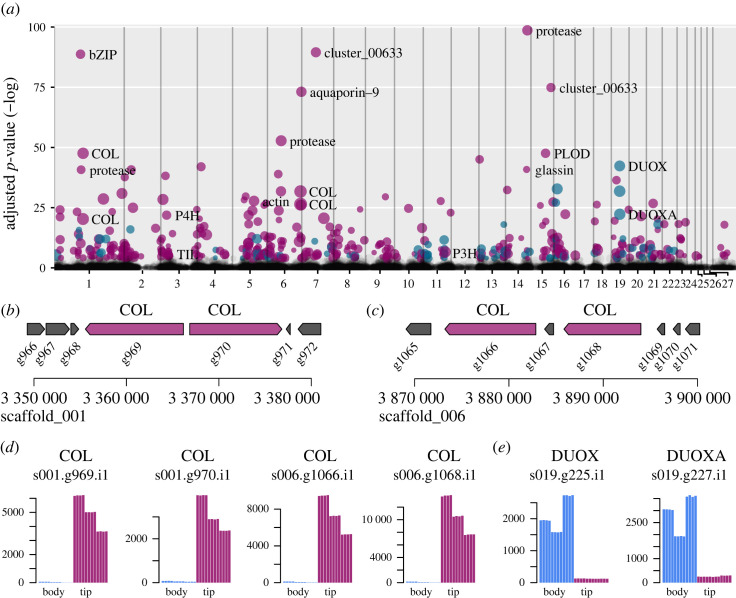
Differentially expressed genes across the genome. (*a*) Manhattan plot of differentially expressed genes, in where *X*-axis positions are based on gene position in the genome, and the numbers below indicate the largest 23 *A. vastus* scaffolds. Grey points are not significant (*p*.adj > 1 × 10^−4^). Purple points are upregulated in the developing osculum and blue points are downregulated. (*b*,*c*) The pairs of collagen loci (in purple) on scaffolds 001 and 006 respectively. Individual exons are not shown for clarity. All other genes are coloured grey. (*d*) Counts of gene expression for the 4 collagen genes, showing significant upregulation in the developing osculum (tip). (*e*) Counts of gene expression of DUOX and the maturation factor DUOXA, showing significant downregulation in the growing osculum.

Collagens are among the most highly expressed genes in general, and four are significantly upregulated in the growing osculum (figure 6*a*,*d*). These are located in two tandem pairs at two loci in the genomes of both species (figure 6*b*,*c*). All three collagen side-chain oxidases (Avas.s015.g314.i1, Avas.s011.g507.i1 and Avas.s003.g260.i1), and several putative proteases (Avas.s001.g887.i1, Avas.s006.g483.i1 and Avas.s014.g646.i1) are also significantly upregulated. These proteins could be involved in modifying newly produced collagen or remodelling existing extracellular matrices. Actins are thought to be involved in the patterning of biosilica deposition in Hexactinellida [7], and of the 43 actin-like genes found in *A. vastus*, one is significantly upregulated (Avas.s005.g703.i1), corresponding to the one identified by Ehrlich *et al*. [7]. The one glassin homologue is significantly upregulated in the developing osculum, suggesting that mineralization also rapidly proceeds at this stage [20]. Other genes were found to be upregulated, including one bZIP transcription factor (Avas.s001.g868.i1) with unknown function, and two members of paralogue cluster 00633, a glass-sponge specific gene cluster with 9 genes of unknown function from *A. vastus*, all of which had 1-to-1 orthologues in *O. minuta*.

Among the most downregulated genes, we found homologues of dual oxidase (DUOX, Avas.s019.g225.i1) and the maturation factor (DUOXA, Avas.s019.g227.i1) (figure 6*a*,*e*). These are tandem genes occurring in a back-to-back gene arrangement, also found in *O. minuta*, the demosponges *A. queenslandica* and *E. muelleri* (electronic supplementary material, figure S10), the human genome (https://www.ncbi.nlm.nih.gov/gene/53905), and the choanoflagellate *S. rosetta*, showing that this gene arrangement predates the origin of the Metazoa and is highly conserved. However, these two genes are, in fact, both found in ctenophores [49], but are not syntenic in either *Hormiphora californensis* or *Mnemiopsis leidyi* suggesting that the gene order was shuffled in the ctenophore lineage. The two genes have a significant expression in pinacocytes of *A. queenslandica* [50], and also in the so-named myopeptidocyte lineage in the demosponge *Spongilla lacustris* [51]. The DUOX protein is a large multi-domain protein with an extracellular peroxidase domain and forms a heterotetramer with DUOXA [52], which may explain why the expression is closely linked in our data, as well as in the two single-cell expression studies referred to above. The protein is thought to be involved in microbial interactions and host defence through the production of reactive oxygen species, but its role in sponges has not been investigated to our knowledge. Other known markers of development in sponges [51] were identified in the genome (3 piwi-like genes, 4 Musashi-like genes) but none were differentially expressed. The *A. vastus* genome also confirms the lack of Wnts, the eponymous ligand of the Wnt signalling pathway, although a large fraction of other proteins involved in the pathway are present (electronic supplementary material, figure S11 and table S10). Overall, the upregulated genes illustrate that mineralization-related genes dominate the expression profile during growth.

### 2.5. The role of proteases and silicateins in the silica skeleton biomineralization

Silicateins, members of the cathepsin protease family, are suggested to be involved in silica mineralization, especially in demosponges [53]. Their precise biochemical role is controversial, as they are proposed to still retain their protease activity [54], catalyse silica deposition through surface residue interactions [6], or serve non-enzymatically as scaffolds [55]. The original description of silicatein from axial filaments of the demosponge *Tethya aurantium* [53] did not include a protease activity assay, explaining instead that it did not display esterase activity (which was not described in their methods). The presence or absence of silicateins across demosponges also does not correspond with the presence of particular spicules either [56], so they are neither necessary nor sufficient for the production of biosilica. Transcriptomic information about sclerocytes is presently minimal in other species. In the single-cell RNAseq dataset for the demosponge *A. queenslandica* [50], none of the cells expressed silicateins (electronic supplementary material, figure S12), suggesting that sclerocytes were not sequenced in this study, or those cells that were present in the sample were inactive. A cluster of cells was identified as sclerocytes in the single-cell RNAseq dataset from the freshwater sponge *Spongilla lacustris* [51]. In these sclerocytes, seven silicatein genes and four cathepsin genes were found to be upregulated, although no other repetitive protein that may function as a scaffolding protein of the amorphous silica was identified. That study made use of a reference transcriptome instead of a genome, meaning that the detected genes could be incomplete, or that genes with low expression may simply be missed. For instance, in our data, the expression of upregulated collagens in the tip was about 100-fold higher than glassin. If sclerocytes were relatively rare and the sclerocyte-specific transcripts were not highly expressed, a single-cell study may simply miss these cells or they could be misassigned to other clusters.

Given that spicules are diverse structures, it is unclear how a diversity of enzymes with the same proposed chemistry would result in the observed variation of spicules across the phylum. This leaves an open question of the actual function of silicateins *in vivo*, and whether this is the same for all sponges. The first possibility is that silicateins and cathepsins both primarily function as proteases and that their role is to make precise cuts in other matrix or mineralization proteins. Extracts of *Suberites domuncula* spicules were demonstrated to have protease activity [54], although as the assay was performed on extracted proteins and not a single recombinant protein, cathepsins may have been present in the extract as well. The extracted glassin protein from *Euplectella aspergillum* was 23 kDa, which was smaller than the predicted protein from the ESTs identified in the same study [9], meaning that some protease must carry out its cleavage. For example, the cleavage motif of human cathepsin L was identified as roughly [V/L]G′G [57], a motif found exactly twice in glassin at the boundaries of its repeat motifs (electronic supplementary material, figure S6). Cleavage at these sites results in a 25 kDa predicted protein consisting of only the repeated segments of both *A. vastus* and *O. minuta* glassins. Indeed, out of the 17 cathepsin genes found in the *A. vastus* genome, four cathepsins were found upregulated and one was downregulated in the developing osculum (electronic supplementary material, figure S13), although this did not include the one cathepsin with serine at the catalytic site (Avas.s001.g1214.i1), as found in silicateins. Potentially, any of these proteases could be involved in the processing of glassin or similar proteins during biomineralization.

The second possibility is that silicateins may have the same chemical role, i.e. that of an enzyme, participating with other enzymes or channels to enrich silica onto a scaffolding protein by dehydrating silicate [58]. The copy number of silicateins could then reflect the need for a large quantity of protein for biochemical or kinetic reasons, such as catalysing a rate-limiting reaction. In such a case, the diversity of spicule shapes would therefore need to come from a diversity of other proteins that are currently unknown in demosponges. Plausibly, this could derive from combinations of repetitive scaffold proteins, similar to glassin or silaffins in diatoms, or the TIL-domain proteins found in this study.

Finally, silicateins may passively form the scaffold itself. In the X-ray crystal structure of *Tethya* silicatein [55], the active site was described as being in a conformation that was predicted to be inactive, unlike the active site of a monomer. Additionally, the first 114 residues (before P115) are absent from the structure, suggesting that these residues were natively cleaved *in vivo*, as other cathepsins autocatalytically process themselves [59] in a similar manner, i.e. at the corresponding position before the same proline. The proposed hexameric structure could then result from the self-assembly of monomers in the filament core. Then, silica can be deposited onto the polymerizing silicateins, possibly through the action of unpolymerized silicatein monomers. If potentially any self-assembling protein structure can serve as a scaffold for silica mineralization (given local thermodynamics favour silica deposition), then this may indicate a mechanism by which glassin functions as a self-assembling scaffold for biomineralization. Because of the mutually exclusive occurrence of putative scaffolding genes for sponge silica mineralization in the demosponges (silicatein) and Hexactinellida (glassin), the processes of silica mineralization in the Silicea *sensu stricto*, i.e. the clade of Demospongiae and Hexactinellida [4], therefore may not be homologous.

## 3. Conclusion

The genome of the reef-building glass sponge *Aphrocallistes vastus* is small compared to other metazoans, and is rich with nested genes, many of which are conserved. Nested genes are a challenge for automatic annotation, and our study has shown that manual genome curation may be important in such cases. Glass sponges (Hexactinellida) are characterized by a unique skeletal architecture made of amorphous silicon dioxide (SiO_2_), and our genomic view on glass sponge biomineralization has revealed the gene activity that is needed for the formation of the massive glass sponge reef structures to elevate themselves above the sea floor. Silica biomineralization is the key trait enabling this growth, and we have shown that mineralization-related genes dominate the expression profile during vertical growth. Interestingly, these genes differ from those identified in other organisms, such as their sister group, the demosponges, indicating the convergence in silica mineralization systems. Despite this progress, the field is still far from identifying the complete gene set for silica mineralization in animals and importantly, how the different shapes of skeletal elements and the unique hexactinellid skeletal architecture are achieved. The additional genomic resources of hexactinellid sponges presented in this study will enable future targeted examination of genes, potentially through gene knockouts, expression knockdowns, or biochemical characterization of groups of proteins. With these tools, future experiments hold promise to deepen our understanding of glass sponge biomineralization and resolve some of the remaining questions in the field.

## 4. Methods

### 4.1. Genomic read data generation and processing

Samples of *Aphrocallistes vastus* (sample GW32145) were collected in 2015 on sponge reefs in the Strait of Georgia (49.039409, −123.320146), British Columbia, Canada. Samples were flash frozen in the field, immediately stored at −80°C and shipped to Munich on dry ice. DNA was extracted using a modified CTAB protocol [60]. Briefly, the sponge tissues were macerated in liquid nitrogen and the resulting powder was digested with proteinase K in a CTAB + PVP buffer. After lysis, polysaccharides were removed using a KoAc precipitation, and the resulting solution was extracted using one volume of chloroform. DNA was recovered from the aqueous phase through isopropanol precipitation and resuspended in water. The extractions were quality controlled in 0.5% agarose gels and quantified using a Nanodrop 1000 spectrophotometer. Only extractions with 260/230 and 280/230 ratios >1.8 were further processed.

We used the ACCEL-NGS 1S Plus DNA library preparation kit (Swift Biosciences, Inc.) to generate shotgun libraries for Illumina sequencing. The library was quantified and quality-controlled on a Bioanalyzer 2100 and paired-end (150 bp) sequenced on an Illumina MiniSeq in high output mode, for a total of 37 815 752 read pairs. All reads were corrected *in silico* with karect [61].

In addition to short genomic reads, we sequenced Oxford Nanopore Technologies (ONT) long reads on PromethION (one R9.4 flowcell) and MinION (three R9.4 flowcells) sequencers using ONTs 1D library preparation kits (SQK-LSK108 for the PromethION and two MinION runs; SQK-LSK109 for the third MinION run) to generate long-insert shotgun genomic libraries. Reads were adaptor-clipped using porechop [62], followed by error correction in LoRDEC [63] with all corrected short reads. Based on mapping to the final assembly (using minimap2 [64]), the average coverage with the combined ONT long reads was 105.9-fold.

To scaffold the assembled contigs, we generated Hi-C libraries using ARIMA Genomics ARIMA-HiC kit following the manufacturer's instructions, starting from flash-frozen sponge tissue powder thawed in 3% fresh formaldehyde for DNA cross-linking. With the resulting Hi-C genomic DNA fragments, we prepared a library using the ACCEL-NGS 1S Plus DNA library preparation kit, which we paired-end sequenced (50 bp) on an Illumina HiSeq 1500.

### 4.2. Transcriptome read data generation and processing

For the reference transcriptome assembly of *A. vastus*, we extracted RNA from flash-frozen tissue (same sample as for genomic DNA isolation) using TRIZOL, quality controlled the extracted RNA on a Bioanalyzer 2100 and prepared a reference, stranded, short-insert library using Lexogen's SENSE mRNA-Seq Library Prep kit. The resulting short-insert transcriptome library was quality controlled and quantified on a Bioanalyzer 2100 and pair-end sequenced (100 bp pairs) on an Illumina HiSeq1500. These reads were combined with reads generated for differential gene expression (DGE) analyses. Sponges for DGE analyses were collected at Fraser Ridge reef (coordinates: 49.155, −123.379) at 170 m depth in the Strait of Georgia (SoG) using a suction sampler on a ROPOS (‘Remotely Operated Platform for Ocean Science’; operated by the Canadian federal government) into the Biobox on the end of the dive, and kept in seawater from depth while retrieving the ROPOS. As soon as the ROV was secured on deck, the tissues were cut from the sponge without removing it from water, and flash frozen in liquid nitrogen. Samples were transported to the laboratory on dry ice. RNA for DGE was extracted from the growing osculum (tip) as well as the main ‘body’ as control (figure 1*a*) using the Single Cell RNA Purification Kit (Norgen Biotek Corp., Thorold, ON, Canada). cDNA libraries were made from 1 μg of RNA (20 ng ul^−1^) with the TruSeq RNA Library Prep Kit v2 (Illumina, Inc., San Diego, CA, USA) by Delta Genomics (Edmonton, AB, Canada). For both body parts, three biological replicates each with four technical replicates (totalling 24 RNASeq libraries) were sequenced in a single lane on an Illumina NextSeq 500 using the NextSeq Series High-Output Kit (Illumina, Inc., San Diego, CA, USA). Between 9.6 and 14.3 million 151 bp non-stranded paired-end reads were sequenced per library (total read-pairs 304.7 M). All RNAseq reads were filtered using the ‘bl-filter-illumina’ tool of the biolite v1 package [65,66]. Reads with an average Phred quality score below 25 were removed. Between 9.1 M and 13.7 M read pairs were kept for non-stranded libraries and 51.4 M for the stranded library. A summary of all *A. vastus* sequenced RNAseq reads is provided in electronic supplementary material, tables S1.

To improve the genome annotation we additionally produced ONT cDNA long reads on an ONT MinION using two R9.4 and one R10 flowcells. The total RNA for the long-read sequencing was extracted from ‘body’ tissue with TRIzol. cDNA libraries were generated using ONT's cDNA-PCR Sequencing Kit (SQK-PCS109). Basecalling was performed on a GPU node of the Leibniz Supercomputing Centre (LRZ) with ONTs basecaller guppy version 3.2.4 (https://github.com/nanoporetech/pyguppyclient). After basecalling, all reads were screened and trimmed from adapters with ONT's pychopper2 (https://github.com/nanoporetech/pychopper), followed by error correction with LoRDEC.

All short- and long-read statistics for *A. vastus* genomic and transcriptomic/DGE data are provided in electronic supplementary material, tables S1.

For additional comparisons on gene content, we also sequenced 14 hexactinellid species collected by ROV (Kiel 6000) during the 2017 RV SONNE cruise SO254 (PoribacNewZ) of the University of Oldenburg and the LMU Munich, Germany and belonging to the same order as *A. vastus*, i.e. Sceptrulophora. Upon collection, fragments of samples were stored in RNAlater solution (Thermo Fisher Scientific) and stored at −80°C until RNA extraction. RNA extraction and library preparation were done as detailed above. The resulting libraries were quality controlled on a Bioanalyzer 2100 and paired-end sequenced (50 bp and 75 bp) in two independent sequencing runs on an Illumina HiSeq1500 or an Illumina NextSeq 500 (see electronic supplementary material, tables S1, for sample and sequencing details).

### 4.3. Reference hexactinellid transcriptome assembly

Assemblies of filtered high-quality reads (pools of multiple libraries if available) of the SONNE expedition libraries as well as the *A. vastus* reference transcriptome were performed on the Leibniz Supercomputing Center of the Bavarian Academy of Sciences and Humanities using the TransPI pipeline v 1.0.0 [67]. For the short SONNE expedition reads, we used 25, 33 and 37 as kmer lengths for the independent runs.

### 4.4. Genome assembly and quality control

Based on lessons learned from previous genome assemblies, we decided to not go for a ‘single assembler trial and error approach on the entire dataset’ to get the best possible assembly. Instead, we performed a ‘multiple assemblers with multiple datasets approach’ (similar to [68]). A total of seven different assemblers were included in the pipeline: FLYE [69], CANU [70], WTDBG2 [71], MINIASM [72], SHASTA [73], RA and RAVEN [74]. Each assembler was run independently on raw as well as LoRDEC corrected long genomic reads. In addition, all assemblers were run independently on different read bins: reads with 5 kb and longer (total number of reads 629 428 or 6.3 Gb), 10 kb and longer (total number of reads 214 971 or 3.38 Gb), and 15 kb and longer (total number of reads 78 474 or 1.73 Gb). After assembly runs, all assemblies were polished with two separate pipelines, hereafter referred to as HYPO and MEDAKA, based on the main ONT long-read polishers used:

(A) HYPO-polishing steps:
  (a) Hypo [75] polishing using LoRDEC-corrected genomic ONT reads
  (b) Racon polishing [76] using karect-corrected genomic paired-end reads
(B) MEDAKA-polishing steps:
  (a) Racon polishing using LoRDEC-corrected genomic ONT reads
  (b) Medaka [77] polishing using LoRDEC-corrected genomic ONT reads
  (c) Racon polishing using karect-corrected genomic paired-end reads.

This resulted in the generation of a total of 84 assemblies. All scripts are provided in the genome repository (https://github.com/PalMuc/Aphrocallistes_vastus_genome).

After polishing, an iterative scaffolding was performed on the 84 assemblies:

(1) corrected long cDNA reads (full set) using L_RNA_SCAFFOLDER [78]
(2) the TransPI reference transcriptome using L_RNA_SCAFFOLDER
(3) all paired-end RNAseq reads using P_RNA_SCAFFOLDER [79,80].

Following scaffolding, haplo-purging was performed on all 84 assemblies using purge_dups [81] to remove sequence redundancy existing due to locally increased heterozygosity.

We evaluated the 84 haplo-purged assemblies based on a series of metrics, including assembly N50 (larger was better), percent difference to predicted assembly size measured by genomescope2 [82] and bbmap [79] (smaller difference was better), average scaffold length (longer was better), number of scaffolds (fewer was better), BUSCO single-copy complete genes (more was better) and RNA and DNA read mapping (higher percent was better). Assemblies were ranked on each of these metrics, and the ranks were weighted in importance (table of relative weights is found in electronic supplementary material, tables S2). Of these, the FLYE-5 kb MEDAKA-corrected assembly scored best across multiple metrics and was selected for refinement steps, including removal of bacterial scaffolds and final scaffolding using HiC data.

Bacteria were identified by two main metrics:

(1) Scaffold GC: all scaffold with a GC content >40% were selected bacterial candidates.
(2) Mapping statistics of RNA (short and long) reads: due to the nature of bacterial transcription process, which does not involve poly-A tailing of mRNAs, as well as a selection for mRNA during the library preparation, bacterial scaffolds should have very low to zero RNA coverage. We created a blobplot (Rscript available in the repository), which combined both metrics in a GUI, that interactively allowed us to identify bacterial scaffolds.

Bacterial scaffolds were removed and the final scaffolding using HiC data was performed:

HiC reads were mapped to the draft assembly using bowtie2 v. 2.3.5.1 [83] and hicstuff v. 2.3.0 [84] with parameters -e DpnII,HinfI –iterative. The assembly was then scaffolded using instaGRAAL v0.1.6 no-opengl branch [84,85] with parameters -l 4 -n 50 -c 1 -N 5 and automatically curated using instaGRAAL-polish.

After removal of 18 scaffolds shorter than 1 kb (9 kb in total), we ended with the final, bacterial-free, 80.32 Mb assembly, containing 186 scaffolds. The average coverage of the assembly was calculated by back-mapping reads.

### 4.5. Manual gene annotation

We developed a manual annotation strategy to combine various annotation tools and then select the best isoform(s) for each gene. Using combinations of different modes or RNAseq datasets, we generated multiple tracks for comparison, including three different runs for BRAKER2 [25], three different runs of StringTie2 [86], and two different runs for Pinfish [87]. We also generated tracks of BLAST matches against SwissProt proteins and against other sponges to enable the assessment of protein completeness relative to the model proteins.

The tracks are provided in the project repository: https://github.com/PalMuc/Aphrocallistes_vastus_genome/tree/main/jbrowse_genome_browser.

#### 4.5.1. Tracks that were used

— **Braker2** tracks:
  (a) BRAKER2_ONT-RNA_augustus.hints.gff3
  (b) BRAKER2_PE-RNA_augustus.hints.gff3
  (c) BRAKER2_ONT-RNA_protein_alignment_gth.gff3
— **Stringtie2** tracks:
  (a) STRG_ONT-RNA_minimap2.gtf
  (b) STRG_PE-RNA_hisat2_non-stranded.gtf
  (c) STRG_PE-RNA_hisat2_stranded.gtf
— **Pinfish** tracks:
  (a) PINFISH_stepwise_corrected-reads_clustered_transcripts.gff
  (b) PINFISH_pipeline_raw-reads_clustered_transcripts.gff
— **BLAST** (homology) tracks:
  (a) SWISSPROT (HUMAN, YEAST, and ARABIDOPSIS)
  (b) SPONGES (AQUE, EMUE)

Beginning from the BRAKER2 gene models, which included 19 317 genes, we then selected the best isoforms that most-agreed with the combined evidence of long-read RNAseq (minimap2), transcript models from the long-read RNAseq (stringtie and pinfish), BLAST matches to other proteins from model organisms (human, *Saccharomyces* yeast and *Arabidopsis*) and from other sponges, and short-read RNAseq coverage.

In total, our annotation resulted in 20 645 genes. Several recurring problems with the gene models suggest potential avenues for the improvement of annotation software. For example, compared to the BRAKER2 models, 1362 genes were fragmented, while only 332 models appeared to be a false fusion of two or more genes.

The fragmented genes were typically identified by homology to other proteins, visible in cases where one gene matches the first half of a homologue, and the neighbouring gene matches the second half. Such genes often had low RNAseq coverage, meaning it was likely to be difficult for any program to generalize a gene-based purely on the RNAseq data, i.e. that some exon junctions had too few reads to confidently link the exons into one gene model. In such cases of low RNAseq coverage, it may be better to increase the relative weight of protein matches.

Conversely, falsely fused genes were usually identified based on RNAseq. Often, one or more long reads would run the two genes together, but where there were few long reads that supported the fused gene compared to having two separate genes, and would usually be at a ratio of the order of 1 : 100. If the cDNA/nanopore output truly reflects the RNA content of a cell, then this suggests that RNA polymerase would erroneously miss the stop signal at a low frequency and make run-on transcripts. When protein matches were available, these would often show two complete matches of the two parts, again arguing that they were indeed two separate and complete genes in very close proximity.

Thus, some parameters for identifying gene models may be optimized:

1) When RNAseq coverage is low, increase the weight of complete protein orthologues from other species.
2) When RNAseq coverage is high, be stricter about the number of links required to join exons or neighbouring genes. This would reduce the number of genes fused at UTRs.
3) For most genes, prioritize a matching homologue covering 90% of the gene model.
4) In the absence of any of those, rely on de novo assembled transcripts, as this may be necessary for multi-domain genes that have variable domains between species.

Additionally, 1050 gene models were removed, while 1318 genes were added.

In total, 3493 models out of 19 578 were modified or removed (17%), showing that the BRAKER2 pipeline (integrating hints from RNAseq) appeared to be correct for the majority of genes, as far as we could detect. Many genes still were difficult to reconcile due to low RNAseq coverage, so it should not be assumed that all genes are correct. Additionally, this approach highlighted loci where misassemblies in the scaffold (possibly due to alleles) had resulted in genes with unreal introns, which included 295 genes that were manually corrected (or invented) in order to make a plausible protein.

Details of the code can be found at the https://github.com/PalMuc/Aphrocallistes_vastus_genome repository.

### 4.6. Phylogeny of glass sponges

To infer a phylogenetic tree of glass sponges, we collected conserved genes identified by the BUSCO analysis across 22 species, 20 glass sponges (the genomes of *A. vastus* and *O. minuta*, the 14 newly generated hexactinellid reference transcriptomes, plus 4 species from [88–90]) and the two demosponges (*A. queenslandica* and *E. muelleri*) as outgroups (data from [88–90]). Where available, BUSCO orthologues were combined across all species using a custom Python script ‘collate_busco_results.py’ to produce 926 FASTA files, 98% of the 954 total BUSCO metazoan orthologues. Each FASTA file was aligned using MAFFT v. 7.487 with default options and the resulting alignments were concatenated into a supermatrix using the script ‘join_alignments.py’ (code at the repository https://github.com/wrf/supermatrix).

We filtered the supermatrix to select for orthologues with at least 50% taxon coverage. Because four taxa had generally very low coverage (*Hyalonema populiferum*, *Walteria leuckarti*, *Farrea similaris* and *Corbitella* spp.), these taxa were prioritized such that at least two of them must be included for each gene. The final, filtered set had 160 partitions and 53k sites. The taxa with the lowest and highest coverage were *Hyalonema populiferum* and *A. vastus* with 21% and 76% occupancy, respectively. Notably, we identified 55 BUSCO genes that were absent from all glass sponges, but were found in either *Amphimedon* or *Ephydatia*, and 18 BUSCOs that were not found in any of the sponges in our dataset. Similarly, 14 BUSCO genes were not found in any medusozoan genome or transcriptome, suggesting that these ‘universal’ genes could be lost in some lineages [91]. The generated supermatrix was used for phylogenetic inference in IQTree2 v. 2.1.2 [92], using the model ‘LG + F + R5’, for 100 bootstrap replicates, and otherwise default parameters.

### 4.7. Analysis of trans-splicing of mRNA

As known from other metazoan phyla, messenger RNAs appear to have a 5′ leader sequence in *A. vastus*. This sequence was identified from the mapping of the long cDNA reads to the genome. Long reads that contain a leader sequence (therefore are putatively complete from 5′ to 3′) will often be unable to map the leader sequence as a true exon. In the BAM file output from minimap2/samtools, this is evident in the CIGAR string of such reads that a segment has been skipped, indicated by the letter ‘S’ at the start or end of the CIGAR string.

The BAM file was parsed with a custom Python script (get_read_skip_from_bam.py) to generate a table of all reads with a ‘skip’ at the beginning or the end of the read. Plotting a histogram of lengths of the skip indicated a local maximum of around 41–45 bp, with a peak at 44 bp (electronic supplementary material, figure S4).

### 4.8. Counting of nested genes

Nested genes were counted using a custom Python script (‘gtfstats.py’ [93]) using option -N. This found nested genes (approx. 12%, 7% of total exonic basepairs) at amounts comparable to other genomes that were annotated using extensive RNAseq libraries. However, the method of annotation matters substantially, as the two placozoans *H. hongkongensis* and *T. adhaerens* have 1 and 2 nested genes, respectively, as the version of the annotation program (AUGUSTUS) used on those two did not allow nested gene predictions by default. Thus, given that the values found in *H. californesis* (ctenophore) and human are both around 10% nested exonic bases, this seems to be a normal value across multiple animal phyla.

### 4.9. Generation of orthologue clusters

We generated orthologue clusters using many publicly available genomes (see electronic supplementary material, tables S5). We focused on mostly invertebrate taxa and some unicellular outgroups, making a total of 35 initial species. Based on BUSCO [94] completeness, 20 of the species were excluded, resulting in 326 130 total proteins for 15 species, of which 6 are sponges (electronic supplementary material, tables S6).

All protein sequences were concatenated into one file and then aligned using DIAMOND [95], with the e-value set to ‘−e 0.01’ instead of 0.001 to allow for more matches between distantly related paralogues, and otherwise default parameters. Matches were filtered using a custom Python script ‘makehomologs.py’. This produced 4 545 978 non-self matches for 202 677 proteins, meaning that around a third of the proteins did not have any match using DIAMOND.

Nodes were clustered using MCL [96], with the inflation parameter ‘−I 1.2’. This gave 33 794 total clusters, of which 12 114 were single-copy in those taxa that were clustered, i.e. not implying that all 15 taxa had a single copy (which was only 48 clusters). As our analysis had included genomes of varied quality as well as transcriptomes, true counts of universal one-to-one orthologues would be difficult to assess from this type of analysis.

### 4.10. Analysis of orthologue clusters

For each cluster, the sequences were aligned with MAFFT v. 7.487 [97] using the options ‘–maxiterate 1000 –genafpair’, and the phylogenetic tree was inferred using FastTree v2.1.11 [98] with the options ‘-wag -gamma’. Protein domains were identified using hmmscan [99] using the ‘PFAM-A’ database. Clusters, protein trees, and domains were viewed in a custom, interactive viewer created using Rshiny [100] for sorting and analysis.

All code can be found on the repository: https://github.com/PalMuc/Aphrocallistes_vastus_genome/tree/main/ortholog_clusters.

### 4.11. Comparisons to *Oopsacas minuta*

During the production of this paper, a genome of another glass sponge, *Oopsacas minuta*, was made available. Data were downloaded from the NCBI Trace archive, at: https://www.ncbi.nlm.nih.gov/Traces/wgs/JAKMXF01.

Raw data were reformatted for standardized analysis following the instructions here: https://bitbucket.org/wrf/genome-reannotations/src/master/jbrowse-tracks/oopsacas/.

The purpose of those steps was to get proteins and the annotation (GFF) file that match the protein accessions reported in the preprint by Santini *et al.* [23], instead of the systematically assigned GenBank ID numbers on the proteins. To do this, the GenBank format was converted to a GFF format. Using the GFF, transcripts were extracted according to the given exons. The transcripts were translated using the custom Python script ‘prottrans.py’.

### 4.12. Comparison of assemblies and genome bulk statistics

Bulk statistics for various genomes were calculated from the assembly file and the annotation GFF using the Python script ‘gtfstats.py’, as done by Francis & Wörheide [93]. We used an extended dataset of 224 genomes across Metazoa, also including the two choanoflagellates *Monosiga brevicollis* and *Salpingoeca rosetta* (electronic supplementary material, tables S7). Additional calculations were done in R. We had reported the ratio of N50 to total length, as this is a more reliable value for indicating how close the N50 value is to the length of an average chromosome. For instance, if a 1 Gb genome had 20 assembled chromosomes and many other short scaffolds, the N50/total may show a value close to 0.05, while the average scaffold length may be substantially shorter due to the many short scaffolds.

### 4.13. Synteny with other sponges

Although the assembly was not at the level of complete chromosomes, it was still contiguous enough to allow for large-scale comparisons of gene order. Comparisons were only made to the other two species with chromosome-level assemblies: *Ephydatia muelleri* and *Oopsacas minuta*. Here we define synteny as blocks of scaffolds or chromosomes that are identical by descent, resulting in orthologous genes at corresponding positions. These positions may subsequently vary due to the insertion of new genes, deletions, or inversions. Dot plots were generated using custom Python and R scripts as done for the genome of the sponge *Ephydatia muelleri* [90] to other metazoan genomes. Briefly, unidirectional DIAMOND/BLASTP hits are plotted to show the positions of the matches on both genomes. When clusters of points appear in a diagonal line on the plot, or more broadly, on the same respective scaffolds, these would be syntenic genes. High-copy-number gene families were excluded from all matches to remove large gene families or transposons that may have parallel expansions in both genomes, and would thereby produce random, spurious matches that are not true orthologues. A cutoff of 50 blast hits was applied for all analyses (using the option ‘−G 50’).

The significance of clusters was calculated as a Fisher's exact test, as done for Srivastava *et al*. [101] and reimplemented in R for Kenny *et al*. [90]. This calculates the probability from the fraction of matches as a 2 × 2 matrix, of the number of matches between two scaffolds (one from each species), number of matches of those scaffolds to all other scaffolds, respectively, and all other matches. As neither genome was assembled to complete chromosomes, often several significant *O. minuta* scaffold matches were found for any *A. vastus* scaffold.

Code can be found at: https://github.com/PalMuc/Aphrocallistes_vastus_genome/tree/main/synteny.

### 4.14. Differential gene expression

For differential gene expression, tip and body transcriptomic reads were mapped to a reference transcriptome using Salmon [102]; the mapping rate was typically around 80%. The resulting (truncated) counts were analysed in R using DESeq2 [103] to determine differentially expressed genes (DEGs) in *A. vastus*' oscula versus body. For these analyses, technical replicates were ‘collapsed’ to their respective biological replicates using the DESeq2 method *collapseReplicates.* For initial surveying, genes were considered differentially expressed if their log-fold change was larger/smaller than 1/−1 and their Benjamini–Hochberg corrected *p*-values were smaller than 0.05. For reporting, we used a stricter cutoff of 10^−4^. Regardless of cutoff, the expression means, *p*-values, adjusted *p*-values and annotations for all genes can be found in electronic supplementary material, tables S9.

Code associated with this analysis can be found at: https://github.com/PalMuc/Aphrocallistes_vastus_genome/tree/main/differential_gene_expression.

### 4.15. Identification of biomineralization genes

Most genes were identified using blastp v. 2.12.0+ [41] or diamond v. 2.0.13 [95]. Queries were: for glassin, the *Euplectella curvistellata* partial glassin genes (NCBI accessions LC010923.1 through LC012028.1 [9]) were used as queries. For carbonic anhydrases, we built on the dataset from Voigt *et al.* [39], which was also used as queries. For cathepsins and silicateins, we built on the dataset from Aguilar-Camacho & McCormack [104]. For bZIP transcription factors, we built on the dataset from Jindrich & Degnan [105]. For DUOX, we built on the dataset from Hewitt & Degnan [49].

## Data Availability

Data and relevant code for this research work are stored in GitHub: https://github.com/PalMuc/Aphrocallistes_vastus_genome and have been archived within the Zenodo repository: https://doi.org/10.5281/zenodo.7970685 [106]. All sequences are deposited in the European Nucleotide Archive (ENA; https://www.ebi.ac.uk/ena) under Bioproject PRJEB61987. The data are provided in electronic supplementary material [107].
